# Spatial characterization of tangle-bearing neurons and ghost tangles in the human inferior temporal gyrus with three-dimensional imaging

**DOI:** 10.1093/braincomms/fcad130

**Published:** 2023-04-19

**Authors:** Theodore J Zwang, Benjamin Woost, Joshua Bailey, Zachary Hoglund, Douglas S Richardson, Rachel E Bennett, Bradley T Hyman

**Affiliations:** Department of Neurology, MassGeneral Institute for Neurodegenerative Disease, Massachusetts General Hospital, Charlestown, MA, USA; Harvard Medical School, Boston, MA, USA; Massachusetts Alzheimer’s Disease Research Center, Charlestown, MA, USA; Department of Neurology, MassGeneral Institute for Neurodegenerative Disease, Massachusetts General Hospital, Charlestown, MA, USA; Department of Neurology, MassGeneral Institute for Neurodegenerative Disease, Massachusetts General Hospital, Charlestown, MA, USA; Department of Neurology, MassGeneral Institute for Neurodegenerative Disease, Massachusetts General Hospital, Charlestown, MA, USA; Department of Molecular and Cellular Biology and Harvard Center for Biological Imaging, Harvard University, Cambridge, MA, USA; Department of Neurology, MassGeneral Institute for Neurodegenerative Disease, Massachusetts General Hospital, Charlestown, MA, USA; Harvard Medical School, Boston, MA, USA; Massachusetts Alzheimer’s Disease Research Center, Charlestown, MA, USA; Department of Neurology, MassGeneral Institute for Neurodegenerative Disease, Massachusetts General Hospital, Charlestown, MA, USA; Harvard Medical School, Boston, MA, USA; Massachusetts Alzheimer’s Disease Research Center, Charlestown, MA, USA

**Keywords:** tau, ghost tangles, inferior temporal gyrus, tissue clearing, spatial mapping

## Abstract

Studies of post-mortem human tissue provide insight into pathological processes, but are inherently limited by practical considerations that limit the scale at which tissue can be examined, and the obvious issue that the tissue reflects only one time point in a continuous disease process. We approached this problem by adapting new tissue clearance techniques to an entire cortical area of human brain, which allows surveillance of hundreds of thousands of neurons throughout the depth of the entire cortical thickness. This approach allows detection of ‘rare’ events that may be difficult to detect in standard 5 micrometre-thick paraffin sections. For example, it is well established that neurofibrillary tangles begin within a neuron, and ultimately, in at least some instances, persist in the brain even after the neuron has died. These are referred to as ‘ghost tangles’, a term that appropriately implies their ‘difficult to see’ ephemeral qualities. We set out to find ghost tangles as one example of the power of the tissue clearance/image analysis techniques to detect rare events, and to learn what happens at the end-point of a tangle’s life history. We were able to identify 8103 tau tangles, 132 465 neurons and 299 640 nuclei in tissue samples from three subjects with severe Alzheimer’s disease (Braak V–VI) and 4 tau tangles, 200 447 neurons and 462 715 nuclei in tissue samples from three subjects with no significant tau pathology (Braak 0–I). Among these data, we located 57 ghost tangles, which makes them only 0.7% of the total tau tangles observed. We found that ghost tangles are more likely to be found in cortical layers 3 and 5 (49/57), with a select few scattered across other layers 1, 2, 4 and 6. This ability to find rare events, such as ghost tangles, in large enough quantities to statistically test their distribution exemplifies how tissue clearing can be used as a powerful tool for studying selective vulnerability or resilience to pathology across brain regions.

## Teaser

Zwang *et al*. use tissue clearing techniques to image and characterize tau tangles, neurons, and nuclei across cortical layers of human inferior temporal gyrus. Imaging large volumes enabled the identification of dozens of ghost tangles, a rare event, and finds that they are more abundant in cortical layers three and five.

## Graphical Abstract

**Graphical Abstract:**
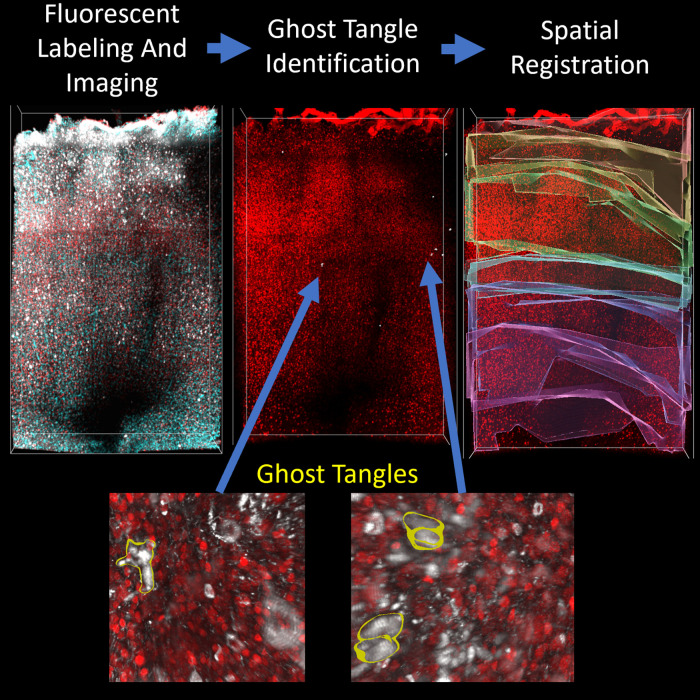

## Introduction

Neurofibrillary tangles of aggregated tau protein are one of the hallmarks of Alzheimer’s disease.^1^ Tau tangles appear in neurons, and mature with age. Visualization of changes in neurofibrillary tangles’ shapes has led to the proposal that their shapes change in a characteristic manner as they mature.^2^ Early stages of tangles or pre-tangles have perinuclear accumulation of tau in addition to a diffuse or granular-staining pattern. Eventually, they encompass the shape of the neuron that they inhabit and move or shrink the nucleus. The final stage of maturity occurs after a neuron dies and leaves behind its extracellular tangle, termed ghost tangles.

Studying these various putative stages of tangle life history would involve finding individual potentially uncommon subclasses of neurons at each stage within the anatomical context of the brain. New methodology that enables the clearing and imaging of large volumes of tissue has promised to allow sampling large populations of individual cells across brain regions with their spatial and structural information intact.^3^ This would enable sampling the diverse heterogeneity in the shape of tau pathology across brain regions and may help uncover mysteries underpinning some cells’ selective vulnerability or resilience to neurodegeneration. However, few experiments have realized the promise of studying cellular distributions in cleared human brain tissue.^4-6^ There are many difficulties that come with scaling up techniques to larger volumes of tissue including inconsistent immunohistochemistry^7^ and historically limited options for analysing large three-dimensional datasets.^8,9^ We focused at first on one of the ‘bookends’ of this process, ghost tangles, since their histological characteristic—the presence of a tangle without an accompanying neuron—is well defined, since they are uncommon in the neocortex and since very little is known even about their spatial characteristics.

We carried out an initial study to analyse the spatial distribution of ghost tangles and other tau pathology at the cellular and regional levels within the inferior temporal gyrus (ITG). The inferior temporal gyrus plays an important role in the mediation of verbal fluency, a cognitive function that is affected early in the onset of Alzheimer's disease.^10^ The ITG is important for semantic language and has important neural connections with the medial temporal cortex structures especially the parahippocampal gyrus.^11^ The ITG is organized into six cortical layers containing different subpopulations of neurons that form distinct connections with other brain regions. The ITG has previously been characterized to receive pathological tau from these connections that begin to form tangles in layers 3 and 5.^12^ This begs the question: does the distribution of mature tangles spatially distribute in a pattern that reflects this early input? We expect that this laminar difference in tau seeding should be reflected in the analysis of other aspects relating to tangle maturity, such as tangle size and the presence of more ghost tangles.

We took advantage of tissue clearing and volumetric imaging to identify and classify many hundreds—of thousands of neurons and tangles across large regions of human brain tissue with their spatial locations intact. To properly quantify the distribution of tau pathology, we used supervised learning software, Ilastik,^8^ to train pixel and object classifiers that identify tau tangles, neurons and nuclei in fluorescence-imaging data. We found that this methodology successfully finds ghost tangles despite their relative rarity, and our results suggest that ghost tangles are preferentially distributed across layers 3 and 5 of the ITG.

## Materials and methods

### Human tissue samples

Fresh frozen human tissue was provided by the Massachusetts Alzheimer’s Disease Research Center (ADRC) with approval from the Mass General Brigham IRB (1999P009556). Three human participants with Alzheimer’s disease and three controls were selected from the Massachusetts Alzheimer's Disease Research Center. Sex, age at death, Braak staging, post-mortem interval and comorbidities are listed in Table 1. Autopsy tissue from human brains was collected at Massachusetts General Hospital, with informed consent of patients or their relatives and approval of local institutional review boards.

**Table 1:** Demographic and characteristics of all subjects used in this study

Sample number	Sex	Age at death	Braak stage	ApoE genotype	Thal stage	Comorbidities	PMI	Cortical thickness (μm)
AD 1 (2399)	M	74	V	4/4	5	CVD	10	2155 ± 44
AD 2 (2302)	F	66	VI	3/3	5	CVD	14	2189 ± 38
AD 3 (2131)	F	≥90	VI	N/A	5	Arteriolosclerosis	5	1827 ± 35
Control 1 (2417)	F	64	I	N/A	0	CVD	18	3452 ± 78
Control 2 (2470)	M	62	0	N/A	1	CVD	17	3020 ± 58
Control 3 (2477)	F	68	I	N/A	0	CVD	20	3005 ± 45

Details include sex (M = male; F = female), age at death, Braak stage, comorbidities (CVD = cerebrovascular disease) and post-mortem interval (PMI; hours). None of the inferior temporal gyrus areas examined contained overt vascular lesions. Cortical thickness was determined by drawing a line from the pial surface to the start of grey matter 10 times at different depths and is represented by the average ± standard error of these measurements.

### Tissue slicing

Brain samples were placed in 4% paraformaldehyde (Thermo Fisher Scientific, cat No. 50980487) for 24 hours at 4°C. Tissue was then rinsed three times with 50 ml phosphate-buffered saline (PBS) for 10 minutes each, then placed in fresh PBS overnight at 4°C and rinsed with fresh PBS. Fixed tissue underwent three rinsing cycles in 10-minute increments using 50 ml of PBS, then were placed in fresh PBS overnight at 4°C. In preparation for tissue slicing, tissue was transferred to individual 35 mm Petri dishes and embedded in a gel block by pouring warm 4% agarose gel solution in PBS (4 g/100 ml) (Promega, cat No. V3121) over the tissue. The gel was then cooled to solidify and cut into a block to provide rigidity for cutting even slices. The tissue was secured on Vibratome (Leica Biosystems, VT1000 S Vibrating Blade Microtome) slide via super gluing the bottom of the agarose block. The vibratome was then used to slice 0.5–1 mm thick sections of tissue. Each slice was then removed from the agarose through gentle manipulation with blunt forceps or paintbrushes and were then placed in crosslinking solution, described below.

### Tissue hydrogel crosslinking

Each slice was incubated with a hydrogel-crosslinking solution containing PBS with 4% (wt/vol) acrylamide (Sigma-Aldrich, ca No. A3553) and 0.25% (wt/vol) VA-044 thermal polymerization initiator (Fisher Scientific, ca No. NC0632395) for 1 day at 4°C to let the molecules diffuse through the tissue. Solutions must be kept cold before and after the addition of VA-044 to prevent premature initiation of polymerization. After incubation with the crosslinking solution for 1 day, the tissue was left in solution and placed in vacuum at 37°C for 3 hours. After polymerization with X-CLARITY polymerization system (Logos Biosystems, South Korea), the slices were rinsed with 50 ml PBS three times over 3 hours. Each slice was then cut into small pieces with width and length at least twice the thickness. This was done to increase the number of conditions that could be tested with small amounts of tissue.

### Delipidation

Tissue was then placed into sodium dodecyl sulphate (Sigma-Aldrich, cat No. L3771) 28.83 g/500 ml PBS-clearing solution supplemented with sodium borate (Sigma-Aldrich, cat No. S9640) on shaker at 100 rpm and 37C for ∼3 days. After delipidation, the brain slices were rinsed with 50 ml PBS five times over 24 hours.

### Immunohistochemistry

Each brain slice was placed in a 2 ml Eppendorf tube that could hold the slice so its large, flat sides could be exposed to solution. PBST (PBS with 0.2% Triton X-100, Thermo Fisher Scientific) was added to just cover the top of the samples (∼500 μl). Tissue was heated to 50°C for 1 hour in PBST and then cooled to room temperature prior to incubation with antibodies. The following conjugated antibodies were then added to the solution containing each tissue slice: phospho-tau Ser202, Thr205 (AT8, 1.6:500, Thermo Fisher, cat No. MN1020) conjugated to Alexa Fluor 647 (Thermo Fisher, cat No. A37573), HuD Antibody E-1 (1.6:500, Santa Cruz Biotechnology, cat No. sc-28299) conjugated to Alexa Fluor 555 (Thermo Fisher, cat No. A37571) and 4′,6-diamidino-2-phenylindole dihydrochloride (DAPI, 1.6:500, Sigma-Aldrich, cat No. 10236276001). Tissue was incubated with primary antibodies for one week at 4°C with gentle shaking. Following incubation, tissue was washed in fresh PBST 3 × 10 min and set on shaker for one week at 4°C with gentle shaking.

### Refractive index matching

After immunohistochemical staining, the samples were incubated with 80% glycerol, 20% deionized water for 24 hours at room temperature with gentle shaking. Samples were then placed on a glass microscope slide with a 3D-printed ring that allows the tissue to remain in a pool of glycerol during imaging. The ring was 3D-printed to match the thickness of the tissue (Formlabs) so a glass coverslip could be placed on top and seal the tissue in the glycerol.

### Imaging

The tissue was then imaged using Olympus Inverted Confocal FV3000 with a 10 × air objective, and multi-region images were stitched together using the microscope software. Some higher resolution images were collected by placing the tissue in a bath of 80% glycerol in a Petri dish and imaged with using a 20 × immersion objective (Zeiss Clr Plan-Neofluar 20x/1.0 Corr) with an inverted Zeiss 980 confocal microscope. Image Z-stacks were then reconstructed and visualized using Imaris microscopy image analysis software.

### Post-imaging analysis

Imaging intensity in each plane was normalized using Imaris’ image-processing toolkit, then data was converted to HDF5 format using Ilastik’s ImageJ plugin plus custom macro to batch convert. Higher resolution images were down-sampled so the pixel sizes were matched across images. Each imaging channel was split into their own individual file. The staining was then segmented using Ilastik’s pixel classifier workflow.^13^ In short, a paintbrush was used to draw over the signal and background to help train the classifier on how to segment each image. All images were then batch processed through the trained pixel classifier, and probability maps were exported as HDF5 format. Pixel probability maps and raw data were loaded into Ilastik’s object classification workflow. 1000 × 1000 pixel regions of each tissue Z-stack were cropped and used to train object classifiers. DAPI and HuD object classifiers were trained by manually classifying objects as noise, single cells or inseparable clusters of cells. Tau object classifiers were trained by manually classifying objects as noise, incomplete circle, complete circle and solid circle shapes. Data was processed as 500 × 500 × 20 blocks and applied over the entire image volumes. Data was exported as object identities and spreadsheets with information about the objects’ classification and characteristics, which were then loaded into MATLAB code to match objects from each channel with colocalized objects.

### Separating objects into cortical layers

Imaris surface generation was used to draw regions around each cortical layer on individual imaging planes within the Z-stack, which was then merged into distinct volumes that contain each cortical layer. These volumes were then used to generate a new channel by masking the pixels contained within each volume and setting them equal to the cortical layer (i.e. pixels in layer 1 = 1, pixels in layer 2 = 2, etc.) and pixels not within a defined layer equal to zero. This channel was then exported as a single multipage tiff stack, which could be loaded into our MATLAB code to identify the cortical layer for each object output by Ilastik.

### Statistical analysis

General statistical analyses were performed using GraphPad Prism 9 and output can be found in Supplementary Table 1, including information on the number of data points used in each test. In all cases, data came from samples from independent subjects (3 AD, 3 Control) using each subject as one data point. One way ANOVA with multiple paired tests were used to determine statistical significance when comparing data across cortical layers or between control and Alzheimer’s disease tissue. Cutoff for statistical significance was *P*-value of < 0.05. Chi-squared test and odds ratio were also calculated in GraphPad Prism.

Linear mixed effect model was fit to data using R. The size of these cells, defined as the HuD stained volume, or the size of neuronal nuclei, defined as DAPI stained volume, was fit to a linear mixed effect model with AT8 status (AT8+ or AT8−) and cortex layer (1–6) as fixed effects, and donor ID as random effects.

### Data availability

All imaging data used in publication is uploaded and publicly available at https://www.ebi.ac.uk/biostudies/bioimages/studies/S-BIAD676. All codes used to quantify data are available and can be accessed publicly at https://github.com/tjzwang/GhostTangles.

## Results

To enable the simultaneous visualization of tau tangles, neurons and other cells in large volumes of human brain tissue, we prepared a library of tissue with variant tissue clearing protocols and found conditions that allow concurrent labelling in human brain tissue as previously described.^7^ We stain the tissue with AT8 antibody that binds to phospho-tau Ser202 and Thr205 of tau protein which is accessible at various stages of tangle maturity, including pre-tangles and mature tangles.^2,14-16^ HuD E-1 antibody binds to the ELAV like RNA-binding protein 1, which localizes to the nucleoplasm, nucleoli and cytosol of neurons.^17,18^ DAPI is a small molecule that binds strongly to adenine–thymine rich regions of double-stranded DNA and localizes to the nucleus of any DNA-containing cell.^19^

**Figure 1:**
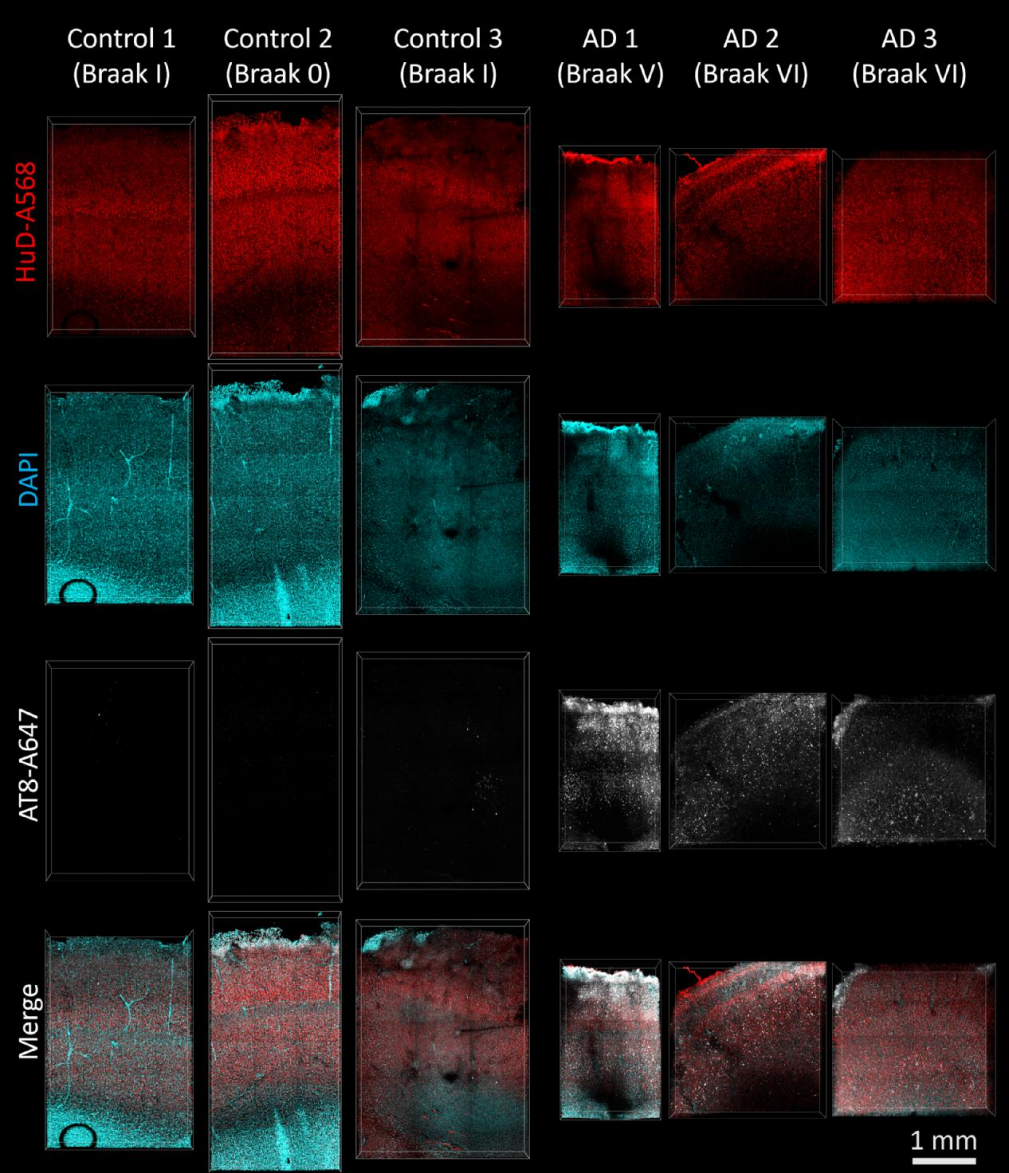
**Immunohistochemistry in cleared human inferior temporal gyrus.** The antibodies HuD-A568 (red) and AT8-A647 (white) as well as DAPI (cyan) were used to label human inferior temporal gyrus tissue from six subjects with different degrees of tau pathology. Additional subject information can be found in Table 1. All tissue samples show clear HuD labelling of neurons and DAPI labelling of nuclei. AT8 staining is significantly more abundant in Alzheimer’s disease cases (Braak V–VI) than in control cases (Braak 0–I).

**Figure 2:**
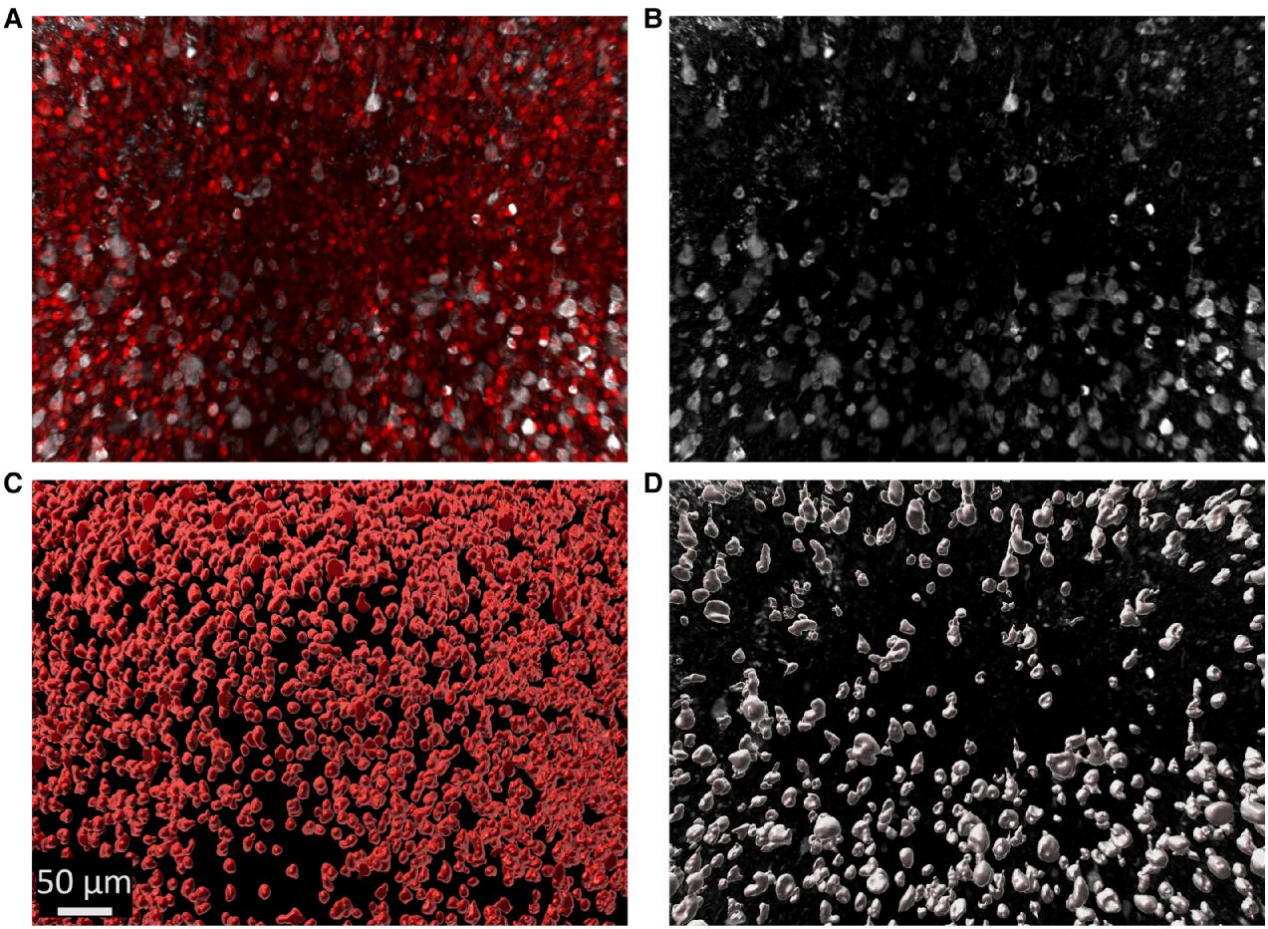
**Semi-automatic identification of neurons and tangles.** (**A**) Top-down view of HuD (red) and AT8 (grey), (**B**) or only AT8 staining through 500 μm. Region is from the same image volume shown in Fig. 1 with Braak stage V. (**C**) Object identities were exported by Ilastik providing information regarding characteristics used for classification, as well as a segmentation mask that was used to visualize and judge the success of registering HuD+ neurons (**D**) and AT8+ tangles. Visualization of segmentation masks were generated using Imaris and using a value of 1 μm to smooth the surfaces.

We stained inferior temporal gyrus tissue samples from six subjects (Fig. 1), three with extensive tau pathology (Braak V–VI) and three with minimal tau pathology (Braak 0–I). Cleared tissue volumes were 0.5–1 mm thick and varied in *x* and *y* dimensions (1–10 mm) to ensure images would contain all cortical layers. This resulted in hundreds-of-thousands of individual cells being imaged in each volume across multiple channels. Attempts to isolate and characterize individual cells within the image volumes using standard threshold or rolling ball algorithms have found some success in past work,^5^ however, we found that it is inadequate for isolating large numbers of densely packed cells or for isolating cells in channels with significant heterogeneity in size and shape, as we see with both tau tangles and neurons across cortical layers. To segment individual cells, tangles or nuclei, we used Ilastik’s pixel classification and object classification workflow (Fig. 2).^8^ This involves supervised assignment of a subset of pixels within the image to teach a classifier to distinguish fluorescent labelling from noise. We trained the classifier on each channel (AT8, HuD and DAPI) separately, then further followed the object classification workflow to separate the individual cells, nuclei or tangles from each as their own object. We were able to identify 8103 AT8+ tau tangles, 132 465 HuD+ neurons and 299 640 DAPI+ nuclei in tissue samples from three subjects with severe Alzheimer’s disease (Braak V–VI) and 4 AT8+ tau tangles, 200 447 HuD+ neurons and 462 715 DAPI+ nuclei in tissue samples from three subjects with no significant tau pathology (Braak 0–I).

**Figure 3:**
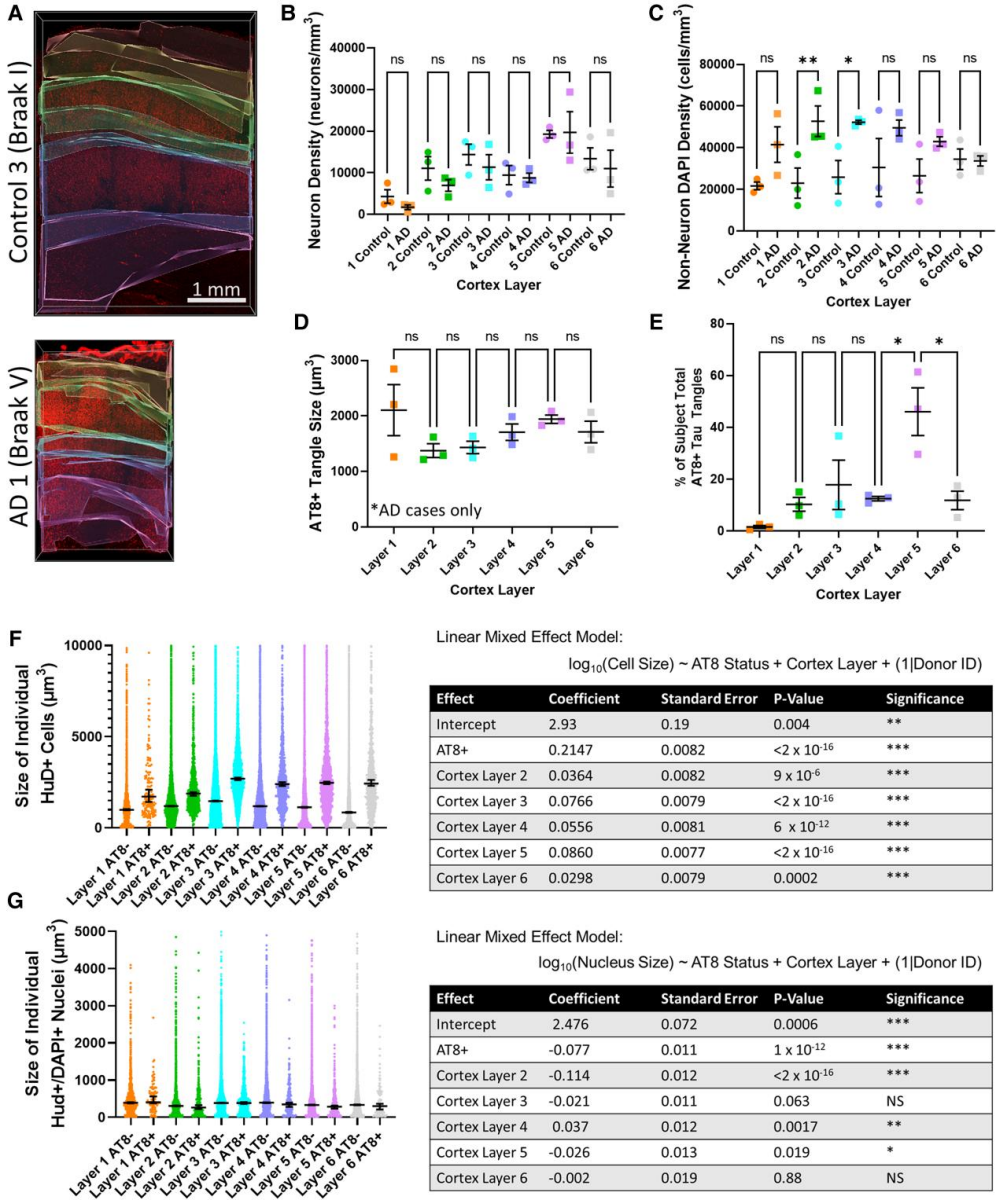
**Comparison of classified cells across cortical layers.** (**A**) Representative examples of segmentation masks that were used to define which part of each image corresponds to different cortex layers. Layers were determined looking at the distribution of neuron sizes (HuD, red) and are artificially coloured from top to bottom: layer 1 = orange, layer 2 = green, layer 3 = cyan, layer 4 = blue, layer 5 = magenta and layer 6 = white. (**B**) Neuron density was calculated by counting individual neurons then dividing by volume to determine the number of neurons present in a 1 mm block of tissue from cortical surface down to white matter. One-way ANOVA showed a statistically significant difference (*P* = 0.0023), however, pairwise *t*-test comparisons between Alzheimer’s disease tissue and control tissue showed no statistically significant difference (*P* > 0.05) likely due to the small sample size and large variability between samples. Each data point represents value from a single individual. *N* = 3 independent samples each for Alzheimer’s disease and control tissue. (**C**) Density of non-neuron DAPI+ cells was calculated similarly and found a statistically significant difference with one-way ANOVA (*P* = 0.018). Statistically significant differences were found in densities between Alzheimer’s disease and control tissue in layers 2 (*P* = 0.005) and 3 (*P* = 0.011) and insignificant differences for layers 1 (*P* = 0.05), 4 (*P* = 0.0589), 5 (*P* = 0.1) and 6 (*P* = 0.93). Each data point represents value from a single individual. *N* = 3 independent samples each for Alzheimer’s disease and control tissue. (**D**) AT8+ tangle size was compared across layers for each of the three Braak V–VI tissue samples tested. There is no significant difference in the sizes of tangles across layers. (**E**) The per cent of each subject’s total tau burden was split across layers (ANOVA *P* = 0.0025). The largest accumulation of tangles is in cortex layer 5, followed by cortex layer 3. A small number of tangles were present in layers 2, 4 and 6, and very few tangles were present in cortex layer 1. Each data point represents value from a single individual. *N* = 3 independent samples each for Alzheimer’s disease and control tissue. (**F**) Comparison between the size of each individual neuron with a tau tangle or without a tau tangle, defined as HuD+/AT8+ and HuD+/AT8− cells, respectively. These data were fit to a linear mixed effect model and showed significant effect on HuD+ cell size from donor, AT8 status and cortex layer. Some cells had sizes larger than the *y*-axis cutoff displayed, which upon inspection were multiple neurons that had been merged into one and were therefore excluded. (**G**) Comparison between the size of each individual neuron nuclei with a tau tangle or without a tau tangle, defined as the total volume of a DAPI+ object that colocalizes with HuD+/AT8+ cells and total volume of a DAPI+ object that colocalizes with HuD+/AT8− cells, respectively. These data were fit to a linear mixed effect model and showed significant effect on DAPI+ nucleus size from donor, AT8 status and cortex layer. DAPI with sizes above 5000 were almost exclusively unsplit clusters of objects and were excluded. Additional statistics information including the number of individual neurons in each layer can be found in the supplemental information. In all plots, statistical significance was represented as: *P* > 0.05 (ns), *P* < 0.05 (*), *P* < 0.01 (**) and *P* < 0.001 (***).

We then sorted the objects based on their cortical layer to quantify their distribution. We used Imaris to manually draw a three-dimensional segmentation mask around each cortical layer (Fig. 3A), then quantified the size and number of neurons, tangles and nuclei across layers. We measured the density of neurons (HuD+) and non-neuronal nuclei (DAPI+/HuD−) across cortical layers (Fig. 3B and C). Although the total number of neurons in the cortex is decreased in more severely affected cases, we found no statistically significant difference in neuron density when comparing layers between control and Alzheimer’s disease tissue, likely due to small sample size and large variability. However, comparing the entire Braak 0 and Braak V–VI tissues, we see a 27.0% ± 7.8% decrease in overall neuron density (two-tailed *t*-test, *P* = 0.04). As expected, we noted an increased density of non-neuronal nuclei among Alzheimer’s disease tissue, especially in cortex layers 2 and 3. There is no significant difference in the sizes of tangles across layers (Fig. 3D). The per cent of each subject’s total tau burden was significantly different across layers (ANOVA *P* = 0.0025) with the largest accumulation of tangles in cortex layer 5, followed by cortex layer 3 (Fig. 3E). A small number of tangles were present in layers 2, 4 and 6 and very few tangles were present in cortex layer 1.

Colocalized staining in each channel was then used to match object IDs from each channel to the same cell using custom code in MATLAB. We used this to determine which HuD-stained neurons have tau tangles and which do not. We fit the size of these cells, defined as the HuD stained volume, to a linear mixed effect model with AT8 status and cortex layer as fixed effects, and donor ID as random effects (Fig. 3F). This modelling shows that the AT8+ neurons have significantly larger cell bodies than AT8− neurons. There was also a significant effect of donor and cortex layer on cell size. In comparison, if we use a similar linear mixed effect model to understand the influence of these effects on the size of neurons’ nuclei, defined by the volume of DAPI+ staining, we see a statistically significant decrease in nuclear volume in AT8+ neurons (Fig. 3G).

**Figure 4:**
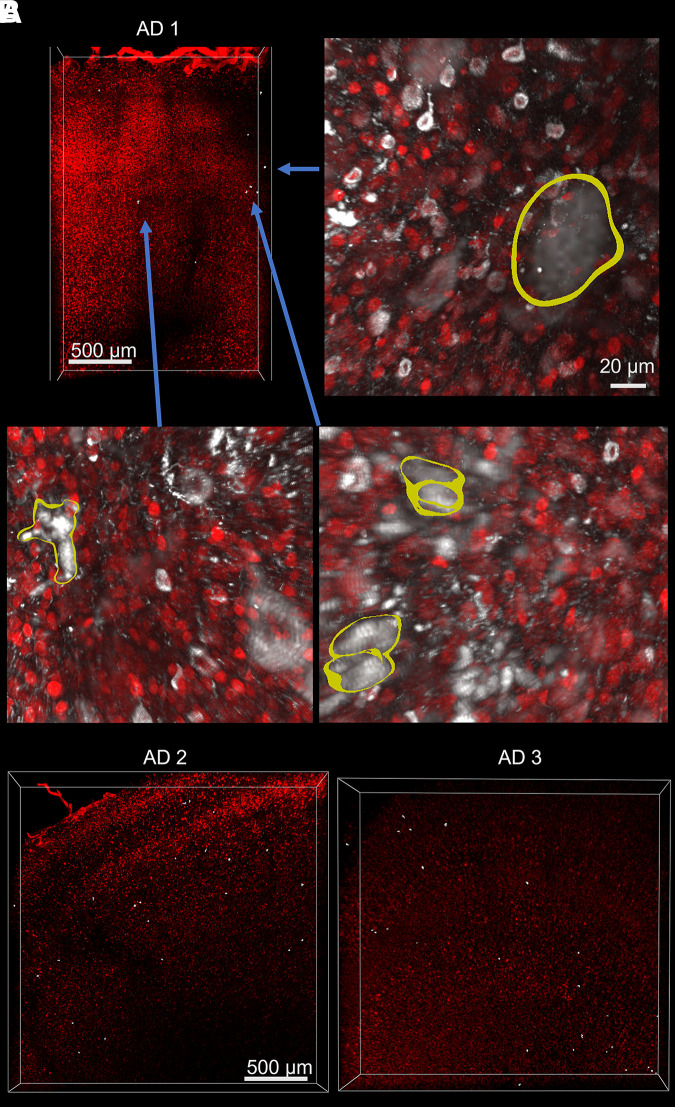
**Location of ghost tangles.** (**A**) Image of inferior temporal gyrus (sample AD 1) from Fig. 1 with HuD (red) and isolated ghost tangles (white). Zoomed-in regions (blue arrow) from tissue in panel **A** show HuD (red), AT8 (white) and with yellow outlining ghost tangles. (**B**) Image of inferior temporal gyrus (samples AD 2 and AD 3) with HuD (red) and location of isolated ghost tangles (white).

We then searched for ghost tangles by using MATLAB to index object IDs that never colocalize with HuD or DAPI along their entire segmented volume. Altogether, we found 57 ghost tangles in Braak 5–6 tissue and 0 ghost tangles in Braak 0–1 tissue (Fig. 4). To ensure correct identification of ghost tangles, manual confirmation was made using an oblique slicer in Imaris to show raw-imaging plane data intersecting with the identified ghost tangle (Video 1). Ghost tangles were exceedingly rare and make up only 57/8107 (0.7%) of all tangles identified in these data. Nearly all the ghost tangles were present in layers 3 and 5 of the ITG (49/57). Analysing their distribution with a Chi-squared test (*P* < 0.0001) or odds ratio together suggests that ghost tangles are 7.9 (95% confidence interval: 3.8 to 16.6) more likely to be found in layers 3 and 5 of the ITG compared to the other layers.

## Discussion

We used tissue clearing techniques to image tau pathology in the inferior temporal gyrus and used supervised machine-learning segmentation to quantify the cytoarchitectural effect of tau pathology on HuD and DAPI positive cells across layers of the cortex. This approach allowed for a three-dimensional visualization and analysis of AT8+ cell bodies including the identification of ghost tangles. This methodology shows that it is possible to use tools like Ilastik to quantitatively analyse large volumes of cleared tissue and find rare cells or features, as well as make comparisons among large amounts of cells segregated by region.

This study suggests that ghost tangles are located primarily in layers 3 and 5 of the inferior temporal gyrus. This aligns with those layers being the first in the inferior temporal gyrus to develop tau pathology.^12^ The relative rarity of ghost tangles and the matching laminar distribution of tangles and ghost tangles is consistent with the assumption that ghost tangles appear at after potentially lengthy maturation period, in concert with tangle formation. These results are consistent with earlier studies of ghost tangles in CA1 of the hippocampus, another area that is affected early in the Alzheimer’s disease process.^2^

The larger cell body of HuD+/AT8+ neurons compared to HuD+/AT8− neurons may result from larger pyramidal neurons typically forming neurofibrillary tangles rather than smaller stellate cells.^12^ An alternate explanation is the morphometric changes that result in swelling of the cell body and shrinking of nucleus that we observe are a neuronal reaction to tau, or a compensatory mechanism, which is consistent with previously reported data that observe shrinking nuclear size in resilient individuals with Alzheimer’s disease pathology.^2,20^ This highlights the possibilities to perform subclass classifications to identify vulnerable versus resilient neuron populations using the techniques described here, along with additional morphological and immunohistochemical markers. However, conclusions made from this study is limited by a low number of samples, and would be improved by the inclusion of an additional cohort of Braak III–IV subject tissue that could provide insight into changes that occur with tangle maturity.

This methodology is a great improvement over simple intensity thresholding or rolling-ball segmentation strategies for quantifying large tissue volumes, but there are still some significant limitations in the ability to segment and classify all cells perfectly. Specifically, we found that choosing thresholds for which pixels merge into an object would require balancing between leaving out some objects that have worse signal-noise-ratio or decreasing the threshold and merging other objects into one. To minimize these issues, we found it to be very important to keep the data being analysed as similar as possible because variations in pixel size and signal-noise-ratio can significantly increase the error rate for Ilastik’s pixel and object classifiers. This is challenging for the accurate assessment of large volumes of tissue, even when cleared, because there may be small inhomogeneities in imaging quality. To reduce variability of images with depth, we found it helpful to normalize the imaging data across each plane in the Z-stack prior to their input into the pixel classification workflow.

Still, there is great promise in using quantitative techniques such as this to characterize the distribution and spatial associations of pathological features. In the future, we hope to expand upon this methodology with other antibodies for pathology, including other phospho-tau antibodies. Understanding the spatial distribution of disease markers will lead to better understanding of the selective vulnerability of brain regions and cell types to neurodegeneration and Alzheimer’s disease.

## Supplementary Material

fcad130_Supplementary_Data

## Abbreviations

AT8 =: monoclonal antibody that binds to phospho-tau (Ser202, Thr205)
DAPI =: 4′,6-diamidino-2-phenylindole
HuD =: monoclonal antibody E-1 that binds to ELAV-like RNA-binding protein 1
ITG =: inferior temporal gyrus
