# Supplementary material for: Spatial characterization of tangle-bearing neurons and ghost tangles in the human inferior temporal gyrus with three-dimensional imaging
Source: Brain Commun. 2023 Apr 19;5(3):fcad130. doi: 10.1093/braincomms/fcad130 (PMC10263274; doi:10.1093/braincomms/fcad130)
Supplement: fcad130_Supplementary_Data [file fcad130_Supplementary_Data.docx]

**Supplementary Table 1** Details from statistical tests in Figure 3. All statistics were calculated using Graphpad Prism 9.5 and provided the following output.

| **Figure 3B** |  |  |  |  |  |  |  |  |
| --- | --- | --- | --- | --- | --- | --- | --- | --- |
|  |  |  |  |  |  |  |  |  |
| ANOVA summary |  |  |  |  |  |  |  |  |
| F | 3.976 |  |  |  |  |  |  |  |
| P value | 0.0023 |  |  |  |  |  |  |  |
| P value summary | ** |  |  |  |  |  |  |  |
| Significant diff. among means (P < 0.05)? | Yes |  |  |  |  |  |  |  |
| R squared | 0.6457 |  |  |  |  |  |  |  |
|  |  |  |  |  |  |  |  |  |
| Multiple comparisons test | Mean Diff. | 95.00% CI of diff. | Below threshold? | Summary | Adjusted P Value |  |  |  |
| 1 Control vs. 1 AD | 2600 | -8297 to 13496 | No | ns | 0.9845 |  |  |  |
| 2 Control vs. 2 AD | 4099 | -6797 to 14996 | No | ns | 0.8737 |  |  |  |
| 3 Control vs. 3 AD | 3047 | -7850 to 13943 | No | ns | 0.966 |  |  |  |
| 4 Control vs. 4 AD | 655.9 | -10240 to 11552 | No | ns | >0.9999 |  |  |  |
| 5 Control vs. 5 AD | -421.3 | -11318 to 10475 | No | ns | >0.9999 |  |  |  |
| 6 Control vs. 6 AD | 2380 | -8516 to 13276 | No | ns | 0.9902 |  |  |  |
|  |  |  |  |  |  |  |  |  |
| Test details | Mean 1 | Mean 2 | Mean Diff. | SE of diff. | n1 | n2 | t | DF |
| 1 Control vs. 1 AD | 4315 | 1715 | 2600 | 3802 | 3 | 3 | 0.6838 | 24 |
| 2 Control vs. 2 AD | 11070 | 6971 | 4099 | 3802 | 3 | 3 | 1.078 | 24 |
| 3 Control vs. 3 AD | 14389 | 11342 | 3047 | 3802 | 3 | 3 | 0.8014 | 24 |
| 4 Control vs. 4 AD | 9454 | 8798 | 655.9 | 3802 | 3 | 3 | 0.1725 | 24 |
| 5 Control vs. 5 AD | 19284 | 19705 | -421.3 | 3802 | 3 | 3 | 0.1108 | 24 |
| 6 Control vs. 6 AD | 13391 | 11011 | 2380 | 3802 | 3 | 3 | 0.626 | 24 |
|  |  |  |  |  |  |  |  |  |
| **Figure 3C** |  |  |  |  |  |  |  |  |
|  |  |  |  |  |  |  |  |  |
| ANOVA summary |  |  |  |  |  |  |  |  |
| F | 2.773 |  |  |  |  |  |  |  |
| P value | 0.0178 |  |  |  |  |  |  |  |
| P value summary | * |  |  |  |  |  |  |  |
| Significant diff. among means (P < 0.05)? | Yes |  |  |  |  |  |  |  |
| R squared | 0.5597 |  |  |  |  |  |  |  |
|  |  |  |  |  |  |  |  |  |
| Multiple comparisons test | Mean Diff. | 95.00% CI of diff. | Below threshold? | Summary | Individual P Value |  |  |  |
| 1 Control vs. 1 AD | -19843 | -39687 to 0.4517 | No | ns | 0.05 |  |  |  |
| 2 Control vs. 2 AD | -29699 | -49543 to -9856 | Yes | ** | 0.005 |  |  |  |
| 3 Control vs. 3 AD | -26372 | -46215 to -6528 | Yes | * | 0.0113 |  |  |  |
| 4 Control vs. 4 AD | -19068 | -38912 to 775.5 | No | ns | 0.0589 |  |  |  |
| 5 Control vs. 5 AD | -16452 | -36295 to 3392 | No | ns | 0.1 |  |  |  |
| 6 Control vs. 6 AD | 861.3 | -18982 to 20705 | No | ns | 0.9294 |  |  |  |
|  |  |  |  |  |  |  |  |  |
| Test details | Mean 1 | Mean 2 | Mean Diff. | SE of diff. | n1 | n2 | t | DF |
| 1 Control vs. 1 AD | 21654 | 41498 | -19843 | 9615 | 3 | 3 | 2.064 | 24 |
| 2 Control vs. 2 AD | 22995 | 52694 | -29699 | 9615 | 3 | 3 | 3.089 | 24 |
| 3 Control vs. 3 AD | 25854 | 52226 | -26372 | 9615 | 3 | 3 | 2.743 | 24 |
| 4 Control vs. 4 AD | 30484 | 49552 | -19068 | 9615 | 3 | 3 | 1.983 | 24 |
| 5 Control vs. 5 AD | 26520 | 42972 | -16452 | 9615 | 3 | 3 | 1.711 | 24 |
| 6 Control vs. 6 AD | 34488 | 33627 | 861.3 | 9615 | 3 | 3 | 0.08958 | 24 |
|  |  |  |  |  |  |  |  |  |
| **Figure 3D** |  |  |  |  |  |  |  |  |
|  |  |  |  |  |  |  |  |  |
| ANOVA summary |  |  |  |  |  |  |  |  |
| F | 1.567 |  |  |  |  |  |  |  |
| P value | 0.2424 |  |  |  |  |  |  |  |
| P value summary | ns |  |  |  |  |  |  |  |
| Significant diff. among means (P < 0.05)? | No |  |  |  |  |  |  |  |
| R squared | 0.395 |  |  |  |  |  |  |  |
|  |  |  |  |  |  |  |  |  |
| Tukey's multiple comparisons test | Mean Diff. | 95.00% CI of diff. | Below threshold? | Summary | Adjusted P Value |  |  |  |
| Layer 1 vs. Layer 2 | 729.6 | -341.7 to 1801 | No | ns | 0.2701 |  |  |  |
| Layer 1 vs. Layer 3 | 672.3 | -399.0 to 1744 | No | ns | 0.3446 |  |  |  |
| Layer 1 vs. Layer 4 | 396.9 | -674.3 to 1468 | No | ns | 0.8077 |  |  |  |
| Layer 1 vs. Layer 5 | 163.5 | -907.7 to 1235 | No | ns | 0.9946 |  |  |  |
| Layer 1 vs. Layer 6 | 394.2 | -677.0 to 1465 | No | ns | 0.8118 |  |  |  |
| Layer 2 vs. Layer 3 | -57.28 | -1129 to 1014 | No | ns | >0.9999 |  |  |  |
| Layer 2 vs. Layer 4 | -332.6 | -1404 to 738.6 | No | ns | 0.8942 |  |  |  |
| Layer 2 vs. Layer 5 | -566 | -1637 to 505.2 | No | ns | 0.5141 |  |  |  |
| Layer 2 vs. Layer 6 | -335.4 | -1407 to 735.9 | No | ns | 0.8911 |  |  |  |
| Layer 3 vs. Layer 4 | -275.4 | -1347 to 795.9 | No | ns | 0.9484 |  |  |  |
| Layer 3 vs. Layer 5 | -508.7 | -1580 to 562.5 | No | ns | 0.6162 |  |  |  |
| Layer 3 vs. Layer 6 | -278.1 | -1349 to 793.2 | No | ns | 0.9463 |  |  |  |
| Layer 4 vs. Layer 5 | -233.4 | -1305 to 837.9 | No | ns | 0.9739 |  |  |  |
| Layer 4 vs. Layer 6 | -2.717 | -1074 to 1069 | No | ns | >0.9999 |  |  |  |
| Layer 5 vs. Layer 6 | 230.7 | -840.6 to 1302 | No | ns | 0.9751 |  |  |  |
|  |  |  |  |  |  |  |  |  |
| Test details | Mean 1 | Mean 2 | Mean Diff. | SE of diff. | n1 | n2 | q | DF |
| Layer 1 vs. Layer 2 | 2104 | 1375 | 729.6 | 318.9 | 3 | 3 | 3.235 | 12 |
| Layer 1 vs. Layer 3 | 2104 | 1432 | 672.3 | 318.9 | 3 | 3 | 2.981 | 12 |
| Layer 1 vs. Layer 4 | 2104 | 1707 | 396.9 | 318.9 | 3 | 3 | 1.76 | 12 |
| Layer 1 vs. Layer 5 | 2104 | 1941 | 163.5 | 318.9 | 3 | 3 | 0.7252 | 12 |
| Layer 1 vs. Layer 6 | 2104 | 1710 | 394.2 | 318.9 | 3 | 3 | 1.748 | 12 |
| Layer 2 vs. Layer 3 | 1375 | 1432 | -57.28 | 318.9 | 3 | 3 | 0.254 | 12 |
| Layer 2 vs. Layer 4 | 1375 | 1707 | -332.6 | 318.9 | 3 | 3 | 1.475 | 12 |
| Layer 2 vs. Layer 5 | 1375 | 1941 | -566 | 318.9 | 3 | 3 | 2.51 | 12 |
| Layer 2 vs. Layer 6 | 1375 | 1710 | -335.4 | 318.9 | 3 | 3 | 1.487 | 12 |
| Layer 3 vs. Layer 4 | 1432 | 1707 | -275.4 | 318.9 | 3 | 3 | 1.221 | 12 |
| Layer 3 vs. Layer 5 | 1432 | 1941 | -508.7 | 318.9 | 3 | 3 | 2.256 | 12 |
| Layer 3 vs. Layer 6 | 1432 | 1710 | -278.1 | 318.9 | 3 | 3 | 1.233 | 12 |
| Layer 4 vs. Layer 5 | 1707 | 1941 | -233.4 | 318.9 | 3 | 3 | 1.035 | 12 |
| Layer 4 vs. Layer 6 | 1707 | 1710 | -2.717 | 318.9 | 3 | 3 | 0.01205 | 12 |
| Layer 5 vs. Layer 6 | 1941 | 1710 | 230.7 | 318.9 | 3 | 3 | 1.023 | 12 |
|  |  |  |  |  |  |  |  |  |
| **Figure 3E** |  |  |  |  |  |  |  |  |
|  |  |  |  |  |  |  |  |  |
| ANOVA summary |  |  |  |  |  |  |  |  |
| F | 7.207 |  |  |  |  |  |  |  |
| P value | 0.0025 |  |  |  |  |  |  |  |
| P value summary | ** |  |  |  |  |  |  |  |
| Significant diff. among means (P < 0.05)? | Yes |  |  |  |  |  |  |  |
| R squared | 0.7502 |  |  |  |  |  |  |  |
|  |  |  |  |  |  |  |  |  |
| Tukey's Multiple Comparisons | Mean Diff. | 95.00% CI of diff. | Below threshold? | Summary | Adjusted P Value |  |  |  |
| 1 vs. 2 | -8.74 | -35.85 to 18.37 | No | ns | 0.8791 |  |  |  |
| 1 vs. 3 | -16.27 | -43.38 to 10.84 | No | ns | 0.3877 |  |  |  |
| 1 vs. 4 | -10.9 | -38.01 to 16.21 | No | ns | 0.7534 |  |  |  |
| 1 vs. 5 | -44.47 | -71.58 to -17.36 | Yes | ** | 0.0014 |  |  |  |
| 1 vs. 6 | -10.26 | -37.37 to 16.85 | No | ns | 0.7944 |  |  |  |
| 2 vs. 3 | -7.53 | -34.64 to 19.58 | No | ns | 0.9301 |  |  |  |
| 2 vs. 4 | -2.16 | -29.27 to 24.95 | No | ns | 0.9998 |  |  |  |
| 2 vs. 5 | -35.73 | -62.84 to -8.623 | Yes | ** | 0.0083 |  |  |  |
| 2 vs. 6 | -1.52 | -28.63 to 25.59 | No | ns | >0.9999 |  |  |  |
| 3 vs. 4 | 5.37 | -21.74 to 32.48 | No | ns | 0.9826 |  |  |  |
| 3 vs. 5 | -28.2 | -55.31 to -1.093 | Yes | * | 0.0398 |  |  |  |
| 3 vs. 6 | 6.01 | -21.10 to 33.12 | No | ns | 0.9719 |  |  |  |
| 4 vs. 5 | -33.57 | -60.68 to -6.463 | Yes | * | 0.013 |  |  |  |
| 4 vs. 6 | 0.64 | -26.47 to 27.75 | No | ns | >0.9999 |  |  |  |
| 5 vs. 6 | 34.21 | 7.103 to 61.32 | Yes | * | 0.0113 |  |  |  |
|  |  |  |  |  |  |  |  |  |
| Test details | Mean 1 | Mean 2 | Mean Diff. | SE of diff. | n1 | n2 | q | DF |
| 1 vs. 2 | 1.56 | 10.3 | -8.74 | 8.07 | 3 | 3 | 1.532 | 12 |
| 1 vs. 3 | 1.56 | 17.83 | -16.27 | 8.07 | 3 | 3 | 2.851 | 12 |
| 1 vs. 4 | 1.56 | 12.46 | -10.9 | 8.07 | 3 | 3 | 1.91 | 12 |
| 1 vs. 5 | 1.56 | 46.03 | -44.47 | 8.07 | 3 | 3 | 7.793 | 12 |
| 1 vs. 6 | 1.56 | 11.82 | -10.26 | 8.07 | 3 | 3 | 1.798 | 12 |
| 2 vs. 3 | 10.3 | 17.83 | -7.53 | 8.07 | 3 | 3 | 1.32 | 12 |
| 2 vs. 4 | 10.3 | 12.46 | -2.16 | 8.07 | 3 | 3 | 0.3785 | 12 |
| 2 vs. 5 | 10.3 | 46.03 | -35.73 | 8.07 | 3 | 3 | 6.261 | 12 |
| 2 vs. 6 | 10.3 | 11.82 | -1.52 | 8.07 | 3 | 3 | 0.2664 | 12 |
| 3 vs. 4 | 17.83 | 12.46 | 5.37 | 8.07 | 3 | 3 | 0.941 | 12 |
| 3 vs. 5 | 17.83 | 46.03 | -28.2 | 8.07 | 3 | 3 | 4.942 | 12 |
| 3 vs. 6 | 17.83 | 11.82 | 6.01 | 8.07 | 3 | 3 | 1.053 | 12 |
| 4 vs. 5 | 12.46 | 46.03 | -33.57 | 8.07 | 3 | 3 | 5.883 | 12 |
| 4 vs. 6 | 12.46 | 11.82 | 0.64 | 8.07 | 3 | 3 | 0.1122 | 12 |
| 5 vs. 6 | 46.03 | 11.82 | 34.21 | 8.07 | 3 | 3 | 5.995 | 12 |
